# Testing the Motor Simulation Account of Source Errors for Actions in Recall

**DOI:** 10.3389/fpsyg.2017.01686

**Published:** 2017-09-28

**Authors:** Nicholas Lange, Timothy J. Hollins, Patric Bach

**Affiliations:** School of Psychology, Plymouth University, Plymouth, United Kingdom

**Keywords:** recall, action memory, enactment, source memory, source monitoring

## Abstract

Observing someone else perform an action can lead to false memories of self-performance – the observation inflation effect. One explanation is that action simulation via mirror neuron activation during action observation is responsible for observation inflation by enriching memories of observed actions with motor representations. In three experiments we investigated this account of source memory failures, using a novel paradigm that minimized influences of verbalization and prior object knowledge. Participants worked in pairs to take turns acting out geometric shapes and letters. The next day, participants recalled either actions they had performed or those they had observed. Experiment 1 showed that participants falsely retrieved observed actions as self-performed, but also retrieved self-performed actions as observed. Experiment 2 showed that preventing participants from encoding observed actions motorically by taxing their motor system with a concurrent motor task did not lead to the predicted decrease in false claims of self-performance. Indeed, Experiment 3 showed that this was the case even if participants were asked to carefully monitor their recall. Because our data provide no evidence for a motor activation account, we also discussed our results in light of a source monitoring account.

## Introduction

Many domestic arguments concern responsibility for actions, such as who last washed up or who left a coffee stain. Lindner et al. (2010) discussed a specific case of memory confusion for actions: the observation inflation effect. In a series of experiments, they reported that participants consistently claimed actions as self-performed when they had merely observed someone else perform those actions. Lindner et al. (2010) argued that observation inflation may emerge from motor simulation during action observation. Accordingly, observing an action engages some of the same neuronal populations as physically executing it (i.e., “mirror neurons,” Brass et al., 2000; Rizzolatti and Craighero, 2004; Bach et al., 2007, 2011; Oosterhof et al., 2013). While the specific function of mirror activation is not clear (Csibra, 1993; Pfeifer et al., 2008; Rizzolatti and Fabbri-Destro, 2008; Hickok, 2009; Bach et al., 2014c), it is normally assumed that observing an action generates an internal replica of the same action, as if it had been self-performed (Grèzes and Decety, 2001; Jeannerod, 2001). Appropriation of observed actions therefore arises because the memory of somebody else’s action not only contains a visual representation of what was observed, but also a motor and proprioceptive representation similar to the memory one has of one’s own actions (Lindner et al., 2010).

Yet, confusion over the source of memories extends beyond memory for actions. Imagination inflation (Garry et al., 1996) is the increased belief in the occurrence of a merely imagined autobiographical event, such as a medical procedure in childhood (e.g., Mazzoni and Memon, 2003). People also confuse the source of two externally experienced events, such as whether they heard about a news story in the paper or on television (Johnson et al., 1993). Most relevant to the observation inflation effect, people have a tendency to claim others’ ideas as their own, an effect labeled unconscious plagiarism or cryptomnesia (Brown and Murphy, 1989; see Perfect and Stark, 2008, for a review). In the prototypical study, participants take turns to generate solutions to a problem. These range from simple verbal fluency tasks (e.g., Brown and Murphy, 1989; Brown and Halliday, 1991) and creativity tasks such as alternate uses for a brick (e.g., Stark et al., 2005; Stark and Perfect, 2006, 2007) to real world problems such as ways of reducing childhood obesity (Perfect et al., 2009). When participants are asked to recall their own solutions, they commonly incorporate solutions generated by their partners, thereby claiming them as their own.

The unconscious plagiarism effect is in many ways analogous to the observation inflation effect, and has been typically explained using the source monitoring framework. Under this account, the source of an item – from whom it originated – is not encoded explicitly alongside an item but inferred at retrieval using qualitative features encoded alongside the item, such as cognitive, affective and perceptual information. Source monitoring failures, such as participants falsely claiming a partner-generated idea as their own, occur because participants did not sufficiently encode those features, are not evaluating them at retrieval, or because features do not clearly distinguish the two sources (Johnson et al., 1993).

The source monitoring framework cannot only account for the unconscious plagiarism error described above, but also predicts that the reverse memory error would occur as well. Indeed, recent work has shown that people do not only “steal” their partner’s ideas, but also “donate” their own ideas. In Hollins et al. (2016a), participants alternated generating solutions to verbal fluency problems. Subsequently, when asked to recall their own ideas, participants showed the well-known unconscious plagiarism effect and included solutions generated by their partner. However, they also produced the opposite error: when asked to recall their partner’s ideas, they mistakenly reported their own ideas. This occurred at about twice the rate that they reported their partner’s ideas in the recall own task (see also Hollins et al., 2016b). Unconscious plagiarism may therefore reflect a more general confusion about the source of memory that occurs when people seek to recall from one source whilst excluding competing sources, in line with the source monitoring framework. This raises the question whether actions are confused in the same way, and if invoking motor system activation is necessary to explain the observation inflation effect.

Thus, the current study tested the claim that motoric encoding of observed actions via motor system activation is responsible for false memories of self-performance (the observation inflation effect). Before we can test the motor activation claim, we need to establish if the observation inflation effect generalizes beyond the paradigm used by Lindner et al. (2010). Lindner et al. (2010)’s paradigm is a variation of the misinformation paradigm (Loftus and Hoffman, 1989) that has previously been used to investigate, for example, the imagination inflation effect (Goff and Roediger, 1998). It consists of three phases. In a first encoding phase, participants are shown action phrases such as “Lift the pen” on a screen. Participants are instructed to read all action phrases and enact a subset using the provided object. In a second encoding phase, some of the previously presented action phrases are presented a second time. Participants in the ‘observation’ condition now watch a video of an actor performing the action phrases they have either read or performed themselves in the first encoding phase. Participants in the ‘re-read’ condition merely read the action phrases a second time. Two weeks later, participants perform a two-phase recognition test. All action phrases from the two encoding phases and some novel action phrases are presented on a screen. For each action phrase, participants decide whether the action phrase was presented at encoding (i.e., is ‘old’) or is novel. When participants judge an action phrase to be ‘old,’ they are asked to decide if they performed the action phrase or merely read it in the first encoding phase. Lindner et al. (2010) were interested in the extent to which different types of additional encoding in the second encoding phase would lead participants to claim they had performed those actions when they had only read them in the first encoding phase. They showed that observation in particular led to increased false claims of performance compared to merely re-reading action phrases. In other words, observation of previously encoded action phrases biased participants’ source judgments in favor of ‘performed’ over ‘read’ responses.

Here, we tested whether observation would still lead to false claims of self-performance if (a) observing someone else perform an action was the only instance of encoding the action (though note that in some variations of the observation inflation paradigm, participants also show observation inflation for novel actions), and if (b) the task at test was to recall self-performed actions rather than make a source-monitoring judgment. If observing actions generally results in false memories of self-performance, we would, as the critical measure, expect participants to falsely recall observed actions as self-performed.

To test the role of the motor trace in false memories of self-performance, we modified the type of actions participants performed. Lindner et al. (2010) asked participants to act out action phrases. Source confusion here may be based on confusion of the verbal in addition to the motor trace. We wanted to limit verbal encoding to minimize source confusion resulting from non-motor traces. Thus, rather than using action phrases, we asked participants to use any part of their body or combination of body parts to take turns performing actions in response to shape cues. While action memory research has largely focused on enactment of action phrases as in Lindner et al. (2010 for review see Engelkamp, 1998; Nilsson, 2000), there are precedents for investigating memory of body movements such as dance moves and movement patterns (Smyth et al., 1988; Foley et al., 1991; Helstrup, 2005). Even though these actions are non-object-directed and unfamiliar, this should not affect potential motor system activation. In fact, the seminal papers revealing motor activation during action observation in humans (e.g., Brass et al., 2000; Stürmer et al., 2000; Calvo-Merino et al., 2004; Chong et al., 2008; Oosterhof et al., 2010) used non-object directed actions, and motoric activation during action observation is typically as least as high for intransitive or unfamiliar actions (e.g., Press et al., 2008; Hétu et al., 2011; Nicholson et al., 2017), which minimize alternative non-motoric encoding strategies such as merely memorizing the objects used, and using them as cues to the actions associated with them (e.g., Decety et al., 1997; Rumiati et al., 2005; Tessari et al., 2007).

Conceptually replicating the observation inflation effect in this novel recall paradigm will allow us to test two predictions of the motor simulation account of false memories of self-performance after observation. First, we know from unconscious plagiarism research that if asked to recall ideas, participants not only steal partner’s ideas but also give away own ideas to a partner (Hollins et al., 2016a,b). If source memory for actions conforms to the same rules, we would expect participants to commit source errors not only when they recall own actions, but to also commit them when they recall actions they observed their partner perform. In other words, in addition to observation leading to false memories of self-performance, we expect that self-performance would also lead to false memories of observation. In fact there is precedence for false memories of performance and observation in source recognition studies in the action memory domain (Hornstein and Mulligan, 2004; Rosa and Gutchess, 2011; Leynes and Kakadia, 2013). In motor simulation views, however, only the former “plagiarism” error is easy to explain. Action mirroring creates a motor trace of the observed action that is added to its visual memory representation. During recall, there is conflict between visual and motoric memory traces, one suggesting observation and the other self-performance, which causes some of the actions to be misattributed. However, such views are hard-pressed to account for the reverse error, where participants misattribute an action to their partner that they had performed themselves. For self-performed actions, both motoric- and visual-memory indicate self-performance; there should thus never be a conflict about the source of a self-performed action. Motor accounts therefore predict a striking asymmetry: while people should readily claim others’ actions as their own, they should very rarely do the reverse. Second, under a mirror neuron network account a disruption of the motor system – due to a secondary task that taxes it – during observation should lead to a reduction in observation inflation. Such effects of motor system load on action observation and interpretation have been demonstrated before, with concurrent motor execution either biasing (e.g., Tipper and Bach, 2008) or disrupting the representation of observed actions or of other visuospatial material (e.g., Quinn and Ralston, 1986; in working memory, Smyth et al., 1988; Smyth and Pendleton, 1989; Lawrence et al., 2001; Can et al., 2017; for a general review see, Schütz-Bosbach and Prinz, 2007; Avenanti et al., 2013). We will therefore test whether impaired motoric encoding of partner’s actions, due to a taxed motor system, results in a reduction in source errors when retrieving own actions.

In sum, the present study will test the following. In Experiment 1 we will determine whether the observation inflation effect reported by Lindner et al. (2010) can be conceptually replicated in a simpler experimental paradigm, which cannot be explained on the basis of a verbal or object-based encoding of the actions. This experiment will also provide a measure of the tendency commit the reverse source error, i.e., to give away own actions. Then in Experiments 2 and 3, we will test the further predictions of a motor simulation account by investigating the impact of concurrent motor and verbal loads during the encoding of partner’s actions on source errors during the recall of own actions.

## Experiment 1

### Method

#### Participants

Thirty-seven members of the public participated for payment of £8. Two participants were excluded from analysis for not attending all sessions. The experiment was reviewed and approved by the Plymouth University, School of Psychology ethics committee. All participants gave written informed consent in accordance with the Declaration of Helsinki.

#### Procedure

**FIGURE 1:**
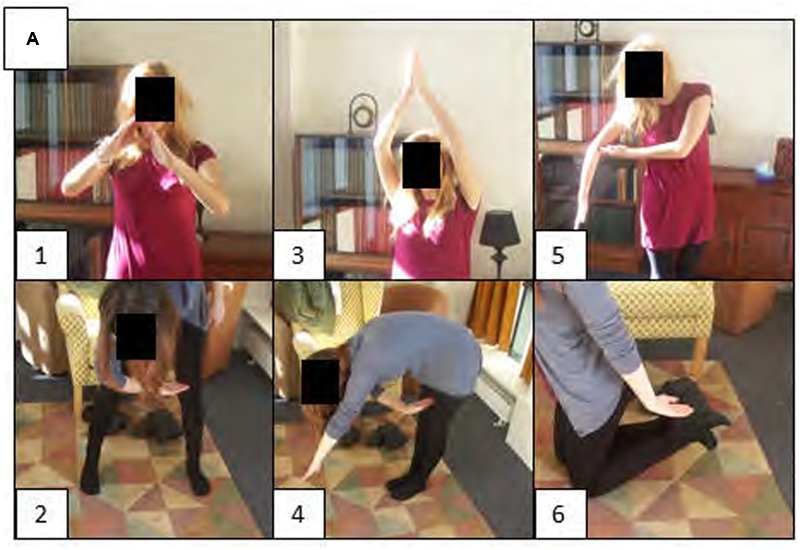
Participants perform actions to represent the shape A.

Participants attended the first session believing they were paired with another naïve participant but in fact were paired with a confederate. Participant and confederate were briefed together by the experimenter and told they would take part in a memory study, with the second session taking place the next day. In the first session, participants completed the generation phase. Participants were instructed that would have to act out a set of 15 shapes (A, C, F, H, I, J, K, L, O, P, T, V, X, △, =), with any part of their body or combination of body parts. The experimenter then demonstrated six different ways a shape can be created with the entire body or combination of body parts for a shape cue not used in the experiment. Participants were cued with a printed label of each shape. Members of the pair took turns generating actions for each cue, interleaving performing and observing actions such that performing an action in response to a cue was followed by observing the other person perform an action in response to the same cue. Each participant generated a total of 3 actions per cue, resulting in 45 performed and 45 observed actions overall (see **Figure 1** for participants acting out the shape A). Participants were told to observe their partners during partner-generation to avoid duplicating exemplars that had already been created for a cue. The participants were explicitly told to produce the shapes so that they seemed correct from their perspective, and ignore how they would look to their partner (i.e., confederate).

Confederates (*n* = 5) were briefed in full about the experiment prior to their participation. They learned up to 15 ways each shape could be made and were instructed to avoid duplicating the participants’ actions.

The naïve participants returned a day later for the test phase. Participants were shown the 15 shape labels one at a time in random order. The Recall own group were asked to re-perform the actions they had performed themselves and were warned not to retrieve actions they saw the other person perform. The Recall partner group was asked to re-perform the actions they had observed their partner perform and were warned not to perform actions they had generated themselves. Participants were asked to re-perform as many exemplars from the appropriate source (self or other) as they could remember for each of the shape cues, working at their own pace.

#### Experimental Design

We manipulated the retrieval task at test in a between-subjects manipulation (Recall own: *N* = 18; Recall partner: *N* = 17). Each action retrieved by participants was coded as a correct recall, a source error (the action was from the correct or incorrect source for the task, respectively) or an intrusion error (the action was not generated at encoding and therefore neither seen nor performed). As we discuss in section “Analytic Approach,” our focus is particularly on the number of source errors participants committed in both retrieval tasks.

#### Action Coding

Photographs were taken of all actions performed during generation and test for both participant and confederate. These were coded by the experimenter (NL), using a coding scheme developed in a pilot study. For each shape between 20 and 40 distinct solutions were identified and assigned categorized numbers. The generation and retrieval phase were then coded separately. Actions were coded as matching the action in the coding scheme when participants performed the exact movement. Crossing one’s forearms to make an X was coded as a different action than crossing one’s arms at the elbows to make an X, for example. It was necessary to distinguish shapes in such a subtle manner, in order to rule out that participants could simply remember in a verbal format how the action were produced (e.g., making an X with the arms). Pilot testing has shown that this task indeed causes participants to perform their shapes repeatedly with the same body parts, so that simple verbal encoding was impossible, or at least very difficult.

To test the reliability of the coding scheme, a subset of the photographs was coded by two independent raters naïve to the purpose of the study and the condition of each participant. The independent raters coded the photos for the first 20 participants, with one rater coding generation phase photographs from the first half and test phase photographs from the second half of those twenty participants, while the other rater coded generation phase photographs from the second half and test phase photographs from the first half of participants. Inter-rater agreement between the experimenter and the two raters was 87 and 91% each, confirming the reliability of the coding scheme. Subsequent analyses were solely based on the experimenter’s judgments.

### Analytic Approach

Each action retrieved by participants was coded as a correct recall, a source error or an intrusion error. **Table 1** shows the frequency of those responses for all experiments. We will report the conventional analyses of the effect of manipulations on the frequency of correct responses, source errors and intrusion errors, with source errors the focus of our interest. However, one concern about source errors in any memory retrieval task is that source errors might either be a genuine memory error (the measure of interest) or simply a guess – an ad-hoc solution generated during the retrieval task – that just happened to be an item also generated at encoding. Source errors (i.e., false source responses to items correctly recognized as old) are therefore often analyzed in relation to false source responses to novel items (i.e., false source responses to items falsely recognized as old) by measuring either the difference or the ratio of both types of errors.

**Table 1:** Full set of mean frequencies of reported responses (SDs in brackets) in Experiment 1 through 3 for both retrieval tasks and all concurrent load conditions.

	Recall own				Recall partner			
	No load	Action planning load	Motor execution load	Verbal load	No load	Action planning load	Motor execution load	Verbal load
**Experiment 1**					
Correct	23.78	–	–	–	15.94	–	–	–
Responses	(3.81)				(3.99)			
Source	8.33	–	–	–	7.65	–	–	–
Errors	(3.50)				(5.30)			
Intrusion	6.33	–	–	–	8.29	–	–	–
Errors	(2.54)				(3.14)			
**Experiment 2**					
Correct	6.89	6.37	6.37	–	4.72	3.50	3.78	–
Responses	(2.56)	(2.39)	(2.06)		(2.24)	(1.29)	(2.53)	
Source	2.63	2.26	2.58	–	3.39	2.83	3.22	–
Errors	(1.54)	(1.37)	(1.84)		(2.00)	(1.82)	(1.83)	
Intrusion	2.42	2.68	2.37	–	2.44	3.33	2.89	–
Errors	(1.89)	(1.83)	(1.64)		(1.79)	(2.83)	(2.42)	
**Experiment 3**					
Correct	8.21	7.21	–	7.00	5.21	3.68	–	3.37
Responses	(2.04)	(1.39)		(2.24)	(2.24)	(2.19)		(1.61)
Source	0.95	1.74	–	1.37	1.37	2.00	–	1.68
Errors	(0.85)	(1.59)		(1.12)	(0.83)	(1.60)		(1.34)
Intrusion	1.89	1.89	–	1.58	2.68	3.05	–	2.74
Errors	(1.52)	(1.63)		(1.77)	(1.45)	(2.44)		(1.56)

*The maximum number of generated items per source and condition are 45 in Experiment 1 and 20 in Experiments 2 and 3.*

Lindner et al. (2010) were interested in the specific effect additional observation had on shifting participants’ response at source test to falsely respond ‘performed.’ To show that the observation inflation effect was not just an effect of guessing, they contrasted the proportion of false ‘performed’ responses after observation in the second encoding phase with the proportion of false ‘performed’ responses for actions that had not been presented in the second encoding phase. Their critical measure was therefore the difference of false ‘performed’ responses when those items had been additionally observed in contrast to when they had not been observed. False ‘performed’ responses to actions that had not been observed presents the baseline of participants giving false ‘performed’ responses irrespective of observing someone else perform those actions. The true effect of observation in their metric is therefore the additional proportion of false ‘performed’ responses observation results in beyond the basic guessing error.

However, this metric cannot be easily transferred to our recall task given the total number of responses at recall differs by participants and observation is the sole encoding instance of an action. We therefore developed a critical measure that would similarly take accidental guessing into account and look at an effect of observation beyond that, in conceptual replication of Lindner et al.’s (2010) metric. We used a Monte Carlo procedure to simulate how many source errors participants would commit if they were guessing and had just generated potential shapes for each symbol “on the fly” during the test phase, rather than genuinely retrieving them from what they had previously either seen or performed. The simulation was based on the distribution of actions generated by participants in the generation phase in response to the shape cues. We simulated the test phase of the experiment for each participant and each shape separately to take into account differences between individual participants, differing frequency profiles for the different shapes, and the typicality of individual items. To achieve this, we used as much of the participant-provided observed data as possible to ensure that the only simulated part of the experiment would be the test phase.

As a first step of the simulation process, we determined frequency norms for the different actions generated for each of the 15 shapes used in the experiment, from all participants who took part in the encoding phase. Participants generated between 20 and 40 different ways of performing each shape across the experiment, with some actions produced more frequently than others. For each shape, we converted those frequency profiles of the different actions into probability distributions, reflecting the relative frequency that a particular action was produced for a given shape. For each shape, the probabilities summed to 1 to represent the entire action space.

We next applied these distributions to the observed test phase for each participant. To simulate a participant’s performance we took the total number of actions they performed (i.e., reported at recall) for each shape in the test phase and randomly selected this number of actions from the overall probability distribution for that shape. This sampling was done without replacement to match the experimental procedure of only retrieving an item once. This provided us with an estimate of which actions would most likely be chosen by a participant if the participant had just generated novel solutions at test, i.e., guessed a number of unique items without memory, under the assumption that these novel solutions at test would follow the same frequency distribution as during the generation phase. We then estimated how many of these novel (simulated) solutions matched this participant’s self-generated actions, matched the actions they observed their partner perform, or were neither seen nor performed by this participant. We repeated the sampling procedure 500 times for each participant to arrive at stable estimates. As with the observed performance, we summed the simulated performance across all shapes. Source errors were now novel (simulated) solutions that happened to be partner actions in the Recall own and self-generated actions in the Recall partner task.

To estimate how many of the observed source errors were the product of guessing we had to scale the simulated performance to the observed performance, based on the number of intrusion errors (actions that were not generated at encoding) committed by participants in the test phase. The assumption here is that intrusion errors must be the result of guessing (e.g., based on how typical or common the actions are), because those actions do not contain source-specifying information. We created the ratio of simulated source errors over all simulated errors for the simulated data for each participant (Eq. 1) and applied that ratio to that participant’s observed data (Eq. 2) to estimate how many source errors we would predict to observe if the participant was guessing given the number of intrusion errors that particular participant committed in the test phase. This gave us the number of predicted source errors, i.e., an estimate of the number of source errors we have to expect if recall was based on the probability of the individual actions, in addition to the number of observed source errors from the experiment.

(1) $$\mathrm{Ratio}=\frac{1}{500}\sum_{i\;=\;1}^{500}\frac{{Source Errors}_{\mathrm{sampled}}}{{Source Errors}_{\mathrm{sampled}}+{Novel actions}_{\mathrm{sampled}}}$$

(2) Source Errorspredicted=Novel actionsobserved*ratio1-ratio

All subsequent analyses we performed were based on these data, with data type (observed, predicted) used as a factor, i.e., in Experiment 1 we can ask whether source errors (for Recall own and Recall partner tasks) exceed the frequency we would expect if participants were guessing. Given our theoretical questions, we will focus on that aspect of the data but will briefly discuss the conventional analyses of the data for a complete account of the experimental results.

### Results

#### Generation Phase

While participants were instructed to avoid duplicating their own or their partner’s actions during generation, some participants still committed such errors. On average, participants duplicated 0.51% (*SD* = 1.22%) of the actions they had already performed themselves and 1.27% (*SD* = 1.81%) of actions performed by the confederate. Confederates never duplicated their own actions and mistakenly duplicated on average 0.57% (*SD* = 1.25%) of the participant’s actions. Partner-duplicated actions were removed from the experiment, and all subsequent analyses were restricted to actions that had only been performed by one person.

#### Test Phase

We will first briefly describe the conventional analyses of the data. Performance at retrieval (correct responses, source errors, intrusion errors) was analyzed with multiple 2 Task (Recall own, Recall partner) ANOVAs. Overall, more items were correctly recalled in the Recall own than Recall partner task, *F*(1,33) = 35.29, *MSe* = 15.21, *p* < 0.001, η^2^ = 0.517. There was no evidence for a difference in the number of source errors reported, *F* < 1, but novel intrusions occurred more often in the Recall partner than Recall own task, *F*(1,33) = 4.15, *MSe* = 8.11, *p* = 0.05, η^2^ = 0.112.

**FIGURE 2:**
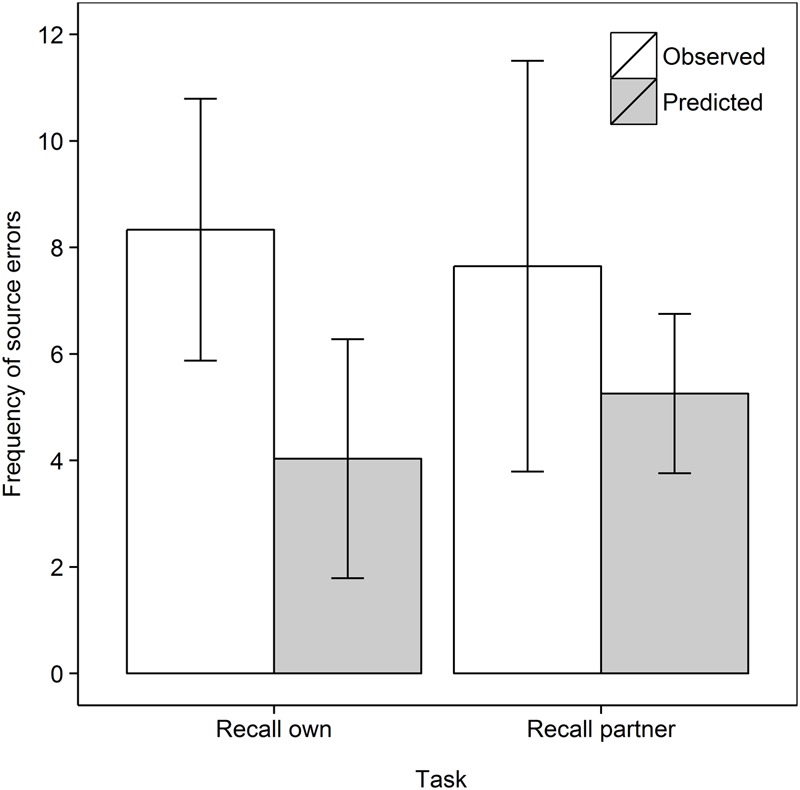
Frequent of observed and predicted source errors in the Recall own and Recall partner task in Experiment 1. The error bars are 95% within-subjects confidence intervals.

Next we turn our attention to the main purpose of our work, and analyze the frequency of source errors observed versus that predicted by guessing, as shown in **Figure 2**. Analyzing the data as a 2 Task (Recall own, Recall partner) × 2 Data type (Observed, Predicted) mixed ANOVA with repeated measures on the second factor, shows no main effect of Task, *F* < 1, but, importantly, a main effect of Data type, *F*(1,33) = 5.64, *MSe* = 12.53, *p* = 0.024, $\eta_{p}^{2}$ = 0.146. Source errors were observed more frequently than predicted, demonstrating the hypothesized observation inflation effect. However, there was no interaction between Task and Data type, *F*(1,33) = 1.03, *MSe* = 10.80, *p* = 0.32, $\eta_{p}^{2}$ = 0.030. In subsequent step-down analyses, we tested whether observed source errors were greater than predicted in each of the two retrieval tasks, that is, whether both the “donating” and “stealing” effects was observed (Perfect et al., 2009; Hollins et al., 2016a,b). Indeed, observed frequencies surpassed predicted frequencies in the Recall own task, *t*(17) = 3.92, *p* < 0.001, *d*_av_ = 1.28 and in the Recall partner task, *t*(16) = 1.84, *p* = 0.042, *d*_av_ = 0.59, both one-tailed.

### Discussion

We successfully conceptually replicated the observation inflation effect of Lindner et al. (2010) in a new paradigm. Using a single encoding phase and non-verbalisable actions, we found false free-recall of observed actions as being self-performed, ruling out that the effect merely reflects an enhanced verbal or object based encoding of the seen actions. Importantly, however, we also observed the opposite effect: the tendency to attribute self-performed actions to a partner. This is in line with reports of participants giving away ideas in the verbal domain (Perfect et al., 2009; Hollins et al., 2016a,b) and action domain (Hornstein and Mulligan, 2004; Rosa and Gutchess, 2011; Leynes and Kakadia, 2013).

This latter effect argues against a simple motor activation account of observation inflation, according to which no confusion should arise for self-performed actions because both visual and motoric memories indicate self-performance. However, while there was no significant difference, **Figure 2** shows that the effect in the Recall own task was greater than in the Recall partner task. This pattern would be in line with predictions of motor theories of observation inflation (e.g., Lindner et al., 2010) while the reverse error may only be an artifact of guessing processes. Thus, while the reverse error exists clearly for verbal material (Hollins et al., 2016a,b), it may not for actions.

Alternatively, the less robust effect in the Recall partner task may emerge from using confederates. Confederates were shown possible actions beforehand and took part in the experiment repeatedly. They may have therefore performed the shapes in a more pronounced and/or prototypical way than naïve participants’. In fact, some naïve participants did report that they suspected they had been paired with a confederate from how the confederate performed the actions. It is therefore possible that knowledge of the confederates or the quality of confederates’ performance would influence the memory performance at test. When looking for own actions, observed actions that were performed quite clearly and confidently may be more strongly represented in memory and intrude as source errors.

To prevent this potential influence, we paired two naïve participants in Experiment 2. If Experiment 2 shows a similar asymmetry, we would have to assume that the difference in effect size is a function of the retrieval task. In contrast, if the difference emerged from the potentially biased action production of the confederates, it should now be eliminated. Experiment 2 also more directly tested the motor simulation account of observation inflation by means of a concurrent load during encoding.

## Experiment 2

According to motor system activation views, observation inflation arises because participants’ motor systems resonate with the actions they observe and produce an internal replica of the action, as if it were self-performed (Craighero et al., 1999, 2002). Occupying the motor system with a secondary motor task should therefore prevent the formation of such traces. Indeed, several studies show that secondary motor task bias or disrupt the formation of action representations or motor plans (cf. Vetter and Wolpert, 2000; Zwickel et al., 2007; Tipper and Bach, 2008; Bach et al., 2014a), and the representation of visuospatial material and its encoding in working memory (e.g., Quinn and Ralston, 1986; Smyth et al., 1988; Smyth and Pendleton, 1989; Lawrence et al., 2001; Can et al., 2017; for a general review see, Schütz-Bosbach and Prinz, 2007). Memories lacking the mirrored motoric information should be less likely to be confused for self-performed actions. In fact, Lindner et al. (2016) recently showed that claims of self-performance after observation were reduced when participants had performed incongruent rather than congruent actions while watching them.

Thus, in Experiment 2, we asked participants to execute simple motor behaviors at the same time as they observed their partner’s actions. This motor performance should interfere with the encoding of motor representations of observed actions, leaving only the visual-perceptual component of the memory trace. We used two types of motor system load to compare against a no load control condition. First, a whole-body motor execution load task was used to directly engage execution-related motor resources, in line with Lindner et al. (2016). Participants were asked to walk in place, swinging their arms, as they watched their partner perform an action. These whole body movements should interfere with the generation of any motor representation of the observed action, irrespective of the body part(s) used. Similar motor tasks have been shown to disrupt recall of action phrases (Saltz and Donnenwerth-Nolan, 1981; Smyth et al., 1988; Helstrup, 2001), the encoding of visuospatial material in working memory (Quinn and Ralston, 1986; Smyth and Pendleton, 1989; Lawrence et al., 2001; Can et al., 2017), or the acquisition of motor skills during mental practice and imitation learning (Bach et al., 2014a). Indeed, prior research has shown that concurrent motor performance affects perception of non-biological and biological action stimuli (for reviews, see Schütz-Bosbach and Prinz, 2007; Avenanti et al., 2013), leading to reductions in subsequent action judgments (Bach and Tipper, 2007; Tipper and Bach, 2008), and interpretations (Vetter and Wolpert, 2000; Hamilton et al., 2004) when produced actions are different from what is currently observed. If motor simulation underlies observation inflation, participants should therefore report fewer partner actions falsely as their own if motoric activation is disrupted due to this secondary task. In contrast, no such difference should be observed if the effects emerge from general source confusion processes outside the motor system.

Second, we used an action planning load task to engage higher-level spatial or action planning resources. We asked participants to remember Corsi-block sequences whilst they watched their partner perform an action. In the Corsi-block task, the experimenter taps a spatial path on a random sequence of blocks arranged on a board. The participant is then asked to reproduce the sequence of taps in the same order. To estimate a participant’s span, the length of the sequence increases until the participant is no longer able to repeat the sequence in the correct order. The length of sequence participants last produced correctly is commonly referred to as participants’ visual-spatial working memory span (Milner, 1971). Action planning is typically assumed to rely on such a visuospatial encoding of the action one intends to perform (Hommel et al., 2001; Hesse and Franz, 2009). It has been argued that mirror neuron activation might not reflect only a motoric encoding of the actions, but also such planning processes (e.g., Csibra and Gergely, 2007; Hickok, 2009; Bach et al., 2014c). Neuroimaging studies show activation during movement planning in the prefrontal, posterior parietal and premotor cortex (Hanakawa et al., 2008; Ikkai and Curtis, 2011), some of the regions implicated in mirror neuron activation (Iacoboni et al., 2001; Koski et al., 2001). The Corsi task has been known for a long time to lead to mutual interference effects in tasks that require visuospatial processing (e.g., Smyth and Scholey, 1994; Della Sala et al., 1999; Vandierendonck et al., 2004; for a review, see Zimmer, 2008).

To test for the possibility that action confusability emerged from such action planning (rather than low-level motor) traces of the actions, we asked participants to remember Corsi-block sequences whilst they watched their partner perform an action. The participant was asked to reproduce the sequence after observing their partner perform an action, meaning the participant had to encode the spatial path tapped by the experimenter as an action intention for later reproduction. Since intentions for future action production are encoded motorically (Brandimonte and Passolunghi, 1994; Freeman and Ellis, 2003; Gallivan et al., 2011) and kept in working memory until actions are executed (Ohbayashi et al., 2003), we expect the motor system to be occupied with that action plan for future performance during observation of partner’s actions.

According to motor simulation views of observation inflation, either or both types of concurrent motor system activity should reduce motor simulation of observed actions and subsequently reduce the number of observed actions falsely recalled as self-performed.

As a direct consequence of the theoretical predictions, the experimental design in Experiment 2 was unbalanced. Concurrent load could only be directly applied to observed actions, not to performed actions. Since concurrent load was applied to blocks of trials, nominally there will be performed actions encoded in Action planning load or Motor execution load blocks, but self-performance of actions always took place without a concurrent load. Any effects of concurrent load on performed actions in those blocks can therefore not be directly an effect of any concurrent load but may be an effect of, for example, encoding context, attention or distraction resulting from switching between target and concurrent load tasks. We will first report the full analysis, looking at all trials of performed and observed actions in the concurrent load blocks. Given the imbalance in the design, we will then specifically analyze the subset of data we manipulated directly and have theoretical predictions about.

### Method

#### Participants

Forty members of the public participated for payment of £12. Three participants did not attend all sessions and their data were excluded from the analysis. The experiment was reviewed and approved by the Plymouth University, School of Psychology ethics committee. All participants gave written informed consent in accordance with the Declaration of Helsinki.

#### Procedure

Participants attended the first session in pairs. Prior to the experiment, each participant’s Corsi-block span was assessed. The length of the tapped sequence was increased up to the point that participants failed to correctly reproduce the sequence twice. Participants’ span was the maximum length of sequence they successfully reproduced twice. Participants were given the same instructions as in Experiment 1 to create exemplars for 15 shape cues with any part of their body or combination of body parts. We asked participants to create 4, not 3 exemplars as in Experiment 1, to compensate for the addition of concurrent load conditions. The 15 cues were split into 3 blocks of five cues each, with a concurrent load (Action planning, Motor execution) added to the action observation trials for two of those blocks (assignment of cues to concurrent load conditions and order of those conditions was counterbalanced across participants), and no load to the remaining block. Participants now performed 20 and observed 20 actions in each of the 3 concurrent load conditions. In the Action planning load condition, participants were shown a Corsi-block sequence at their span prior to observing their partner perform an action, then asked to reproduce the sequence after the observation. In the Motor execution load condition, participants were asked to walk in place, with exaggerated movement of both arms and legs, as they observed their partner. Performance of own actions always took place under no load.

Participants returned to retrieve either their own or their partner’s actions the next day, with the retrieval task identical to Experiment 1. Responses were scored and analyzed as in Experiment 1.

#### Experimental Design

As in Experiment 1, we manipulated the retrieval task at test between-subjects (Recall own: *N* = 19; Recall partner: *N* = 18). Additionally, we manipulated the concurrent load during observation within-subjects (No load, Action planning load, Motor execution load for 5 cues each). Thus in the Recall own task, participants recalled actions they had performed themselves without a secondary load, while avoiding reporting actions they had observed under no load, an action planning load and a motor execution load. In the Recall partner task, participants recalled actions they had observed under no load, an action planning load and a motor execution load, while avoiding reporting actions they had performed under no load. Since we manipulated concurrent load only during observation of actions, we will focus on the effects of concurrent load on the false retrieval of observed actions in the Recall own task.

### Results

#### Generation Phase

Concurrent load had no impact on the tendency for participants to repeat their own actions (No load: *M* = 5.13%, *SD* = 5.75%; Action planning load: *M* = 5.54%, *SD* = 7.42%; Motor load: *M* = 6.35%, *SD* = 6.11%), *F* < 1. However, concurrent load did influence the tendency to duplicate a partners actions (No load: *M* = 9.73%, *SD* = 6.25; Action planning load: *M* = 13.78%, *SD* = 7.75%; Motor load: *M* = 12.16%, *SD* = 6.93%), *F*(2,72) = 3.37, *MSe* = 1.83, *p* = 0.040, $\eta_{p}^{2}$ = 0.086, (with Bonferroni-adjustment, none of the individual pairwise comparisons differed significantly). For the analysis of retrieval performance, only those items that had only been performed by one of the participants in the pair were included.

#### Test Phase

Performance at retrieval (correct responses, source errors, intrusion errors) was analyzed as multiple 2 Task (Recall own, Recall partner) × 3 Concurrent load (No load, Action planning load, Motor execution load) ANOVAs with repeated measures on the second factor.

Correct recall was higher in the Recall own than Recall partner task, *F*(1,35) = 17.64, *MSe* = 3.39, *p* < 0.001, $\eta_{p}^{2}$ = 0.335. There was a main effect of concurrent load, *F*(2,70) = 3.49, *MSe* = 2.34, *p* = 0.036, $\eta_{p}^{2}$ = 0.091, with Bonferroni-adjusted pairwise comparisons not showing a significant difference between concurrent load conditions, though testing the control condition against the average of the two load conditions shows that correct recall was lower when actions were observed under load, *F*(1,35) = 6.29, *MSe* = 23.95, *p* = 0.017, $\eta_{p}^{2}$ = 0.152. The interaction was not significant, *F* < 1.

For source errors, there were no significant effects of Task, *F*(1,35) = 2.10, *MSe* = 1.90, *p* = 0.16, $\eta_{p}^{2}$ = 0.057, or Concurrent load, *F*(2,70) = 1.27, *MSe* = 1.7, *p* = 0.29, $\eta_{p}^{2}$ = 0.035, nor was there a significant interaction, *F* < 1. Similarly, intrusions errors did not show main effects of Task, *F* < 1, or Concurrent load, *F*(2,70) = 1.41, *MSe* = 2.22, *p* = 0.25, $\eta_{p}^{2}$ = 0.039. There was no significant interaction, *F* < 1.

As in Experiment 1, we will now turn to the comparison of the rates of source errors observed versus predicted by guessing. Participants generated fewer items per condition in Experiment 2 than Experiment 1. This resulted in fewer errors in absolute terms, but the error rates were equivalent in both experiments with 19% of partner actions and 17% of self-generated actions reported as source errors in Experiment 1 compared to 15 and 19%, respectively, in Experiment 2.

**FIGURE 3:**
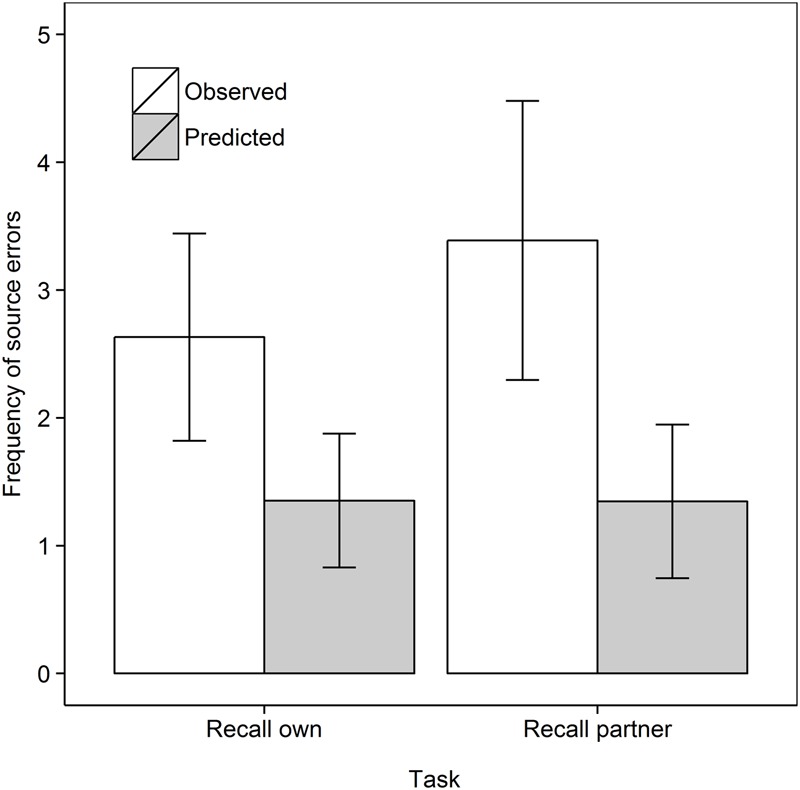
Frequency of observed and predicted source errors in the Recall own and Recall partner task in Experiment 2 for the no concurrent load condition. The error bars are 95% within-subjects confidence intervals.

We first compared source errors in the Recall own and Recall partner task as in Experiment 1, to test the prediction of the motor simulation account that more source errors would be made when recalling own rather than partner actions. Since concurrent load was manipulated only when participants observed their partner’s actions, only the no concurrent load condition was used in this comparison. A 2 Task (Recall own, Recall partner) × 2 Data type (Observed, Predicted) mixed ANOVA with repeated measures on the second factor did not reveal a main effect of Task, *F*(1,35) = 1.01, *MSe* = 2.59, *p* = 0.32, $\eta_{p}^{2}$ = 0.028. As in Experiment 1, source errors were observed more frequently than predicted from guessing, *F*(1,35) = 30.46, *MSe* = 1.67, *p* < 0.001, $\eta_{p}^{2}$ = 0.465. The interaction was not significant, *F*(1,35) = 1.61, *MSe* = 1.67, *p* = 0.21, $\eta_{p}^{2}$ = 0.044. Step-down analyses showed that observed errors surpassed predicted errors in both retrieval tasks, see **Figure 3**, but the effect was smaller in the Recall own task, *t*(18) = 3.15, *p* = .003, *d*_av_ = 0.98, than the Recall partner task, *t*(17) = 4.58, *p* < 0.001, *d*_av_ = 1.26, both one-tailed. This means that the higher rate of errors in the Recall own task in Experiment 1 was not replicated here with naïve participants.

**FIGURE 4:**
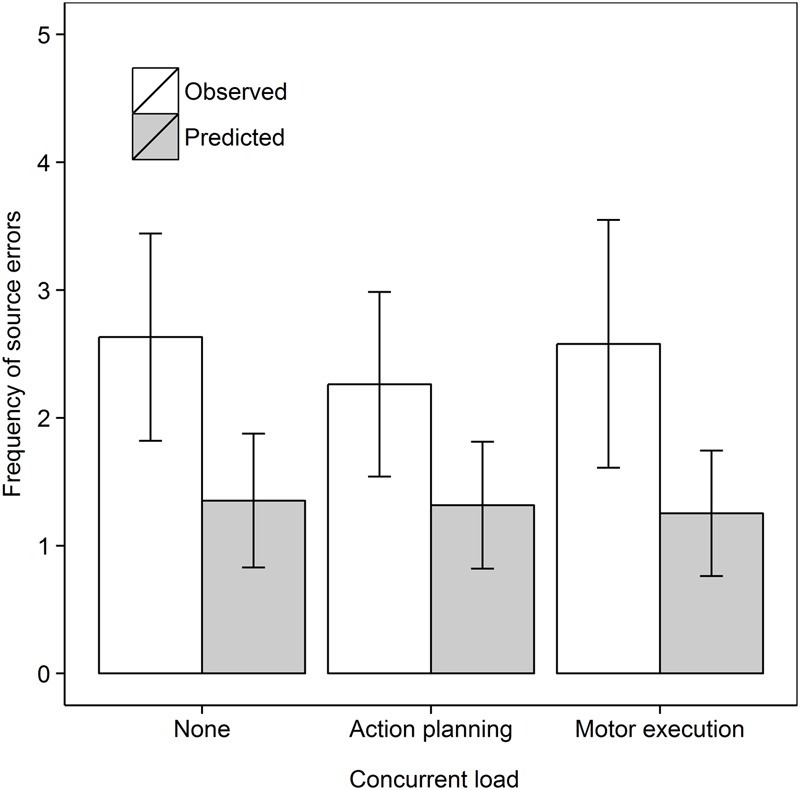
Frequency of observed and predicted source errors in the Recall own task in Experiment 2 for all concurrent load conditions. The error bars are 95% within-subjects confidence intervals.

We then tested whether concurrent (action planning or motor execution) load would reduce source errors in the Recall own task, as shown in **Figure 4**. We analyzed the data for the Recall own group with a 2 Data type (Observed, Predicted) × 3 Concurrent load (No load, Action planning load, Motor execution load) repeated measures ANOVA. As before, source errors were observed more frequently than predicted from guessing, *F*(1,18) = 22.57, *MSe* = 1.77, *p* < 0.001, $\eta_{p}^{2}$ = 0.556. There was no evidence for an effect of Concurrent load nor was there a significant interaction, both *F*s < 1. In subsequent step-down analyses, we tested whether observed source errors were greater than predicted in every concurrent load condition. Observed frequencies significantly surpassed predicted frequencies in the Control, *t*(18) = 3.15, *p* = 0.003, *d*_av_ = 0.98, Action planning load, *t*(18) = 2.73, *p* = 0.007, *d*_av_ = 0.81, and Motor execution load condition, *t*(18) = 2.55, *p* = 0.010, *d*_av_ = 0.91, all comparisons one-tailed.

### Discussion

Experiment 2 replicates the results of Experiment 1. Both observation inflation and the reverse error of falsely recalling self-performed actions occurred more frequently than expected from guessing. If anything, source errors were more frequent in the Recall partner than the Recall own task. This suggests that the smaller effect in the Recall partner task in Experiment 1 was due to the use of confederates. Even though the difference was not significant, it is in line with our prior work in the verbal domain that has similarly shown higher rates for giving away compared to stealing ideas (Hollins et al., 2016a,b).

This pattern of bidirectional source errors in Experiment 2 is not consistent with a motor simulation account of observation inflation. Motor traces generated during action observation would only predict observation inflation, not giving away self-performed actions to the partner. In addition, such views would predict that motor load during action observation should lead to fewer of those actions being falsely retrieved as self-performed, as own motor execution should interfere with generating motoric memory traces. We found no evidence for this account. Neither the motor load, nor the action planning load, decreased source errors. This was not because source errors were at floor. Source errors surpassed predicted frequencies in each load condition quite substantially (*d* > 0.81 in all conditions).

Because we did not observe an effect of concurrent load, it is possible that the manipulation we used had no effect on encoding of observed actions. Note though that similar tasks have been used before to interfere with motoric encoding of observed actions (for reviews see Schütz-Bosbach and Prinz, 2007; Avenanti et al., 2013). Indeed, we did see that concurrent load increased partner-duplications at generation, consistent with its demonstrated effect on working memory (Quinn and Ralston, 1986; Smyth and Pendleton, 1989; Lawrence et al., 2001; Can et al., 2017), and decreased correct recall of partner actions [this simple effect was marginally significant, *F*(2,34) = 3.17, *MSe* = 2.33, *p* = 0.055, $\eta_{p}^{2}$ = 0.157]. This means, while there was no evidence for the predicted effect of the manipulation on source errors in the Recall own task, the manipulation did affect participants’ performance in the experiment overall.

Another possibility is that the free report task we chose as the memory test cannot clearly reflect an effect of the manipulation on source confusion specifically. To make source judgments in a free recall task, participants first have to generate an action and then decide to report or withhold that action from report, depending on whether the inferred source matches the task requirement (recall own or recall partner). Participants’ report therefore conflates generation of solutions and source decisions about these solutions, both of which can be separately affected by experimental manipulations. It is therefore possible that participants in a free report simply neglect the source of retrieved actions and report all actions that come to mind without engaging in explicit monitoring of the source. Even though our calculations suggest that guessing cannot account for the source errors we observed, forcing participants to consider items more carefully may reveal an effect of the concurrent load manipulation on observation inflation. The retrieval task in Experiment 3 was therefore changed to instruct participants to inspect every action they reported at retrieval for source-appropriateness.

A third possibility is that the cognitive load introduced by the secondary task affected participants’ ability to sufficiently encode the actions they observed. In that case, it would be plausible that such resource depletion would increase source errors while the motor component of the secondary task prevented motor simulation and decreased source errors. In that case, we would have expected to see that source errors were in total lower in the motor execution than the action planning load condition. We found no evidence for that. In Experiment 3, we will test this possibility explicitly and introduce a non-motor task with high cognitive load to compare against the action planning load.

## Experiment 3

Experiment 2 did not show the reduction in source errors predicted by a motor simulation account of observation inflation. It is, however, possible that participants simply neglected to consider the source of actions at retrieval and simply reported everything that came to mind as they completed the standard free report task. The retrieval task in Experiment 3 was therefore separated into two separate stages, following an extended recall procedure developed by Bousfield and Rosner (1970), and more recently used by Kahana et al. (2005) and Hollins et al. (2016a). As in the these tasks, we asked participants to perform all actions that came to mind when asked to recall either own or partner actions. For each action performed, participants were then explicitly asked to consider its source carefully and to decide whether or not it was compliant with their retrieval goal (i.e., to recall their own or their partner’s actions). The actions that participants reported are therefore only those they had explicitly attributed to the required source.

Additionally, we replaced the motor execution load from Experiment 2 with a verbal load task to be able to pinpoint a specific motor load effect compared to a generic cognitive one. While a comparison of motor execution to verbal load may be interesting, both loads differ in their cognitive difficulty, confounding difficulty and modality-specifics. The Action planning load task, on the other hand, should be of comparative task difficulty to the verbal load task for participants since they are tested at span if both cases. If source errors are due to motor planning processes, then we would expect to see a reduction of false reports of partner actions as self-performed only under concurrent action planning but not concurrent verbal load. In contrast, if they emerge from a more general source, both loads should affect source errors equally.

### Method

#### Participants

Forty-two members of the public participated for payment of £12. Four participants did not attend all sessions and their data were excluded from the analysis. The experiment was reviewed and approved by the Plymouth University, School of Psychology ethics committee. All participants gave written informed consent in accordance with the Declaration of Helsinki.

#### Procedure

Prior to the experiment, each participant’s Corsi block-tapping span and forward digit span were assessed. The Action planning load condition was identical to Experiment 2. The Motor execution load condition from Experiment 2 was replaced by a Verbal load condition. Participants heard a sequence of digits at their individual span prior to observing their partner perform an action and were asked to reproduce the sequence after their partner completed their action. Concurrent load was only administered during observations of partner actions, not during execution of own actions. As in Experiment 2, participants generated 4 exemplars each in response to 15 shape cues split over 3 concurrent load conditions. Participants returned the next day individually for an extended recall task. They were instructed to retrieve and re-perform either their own actions or those they had observed their partner perform the previous day. They were told that, as they tried to remember their own (or their partner’s) actions, other actions may come to mind such as their partner’s actions when they had to remember their own actions, or entirely new ways of performing each shape. They were encouraged to perform everything that came to mind as they tried to remember their own (or their partner’s) actions, and to indicate verbally for each action whether it was a target action or not. They were not instructed to search their memory for actions from both sources, nor to generate entirely new actions.

#### Experimental Design

As in Experiment 1, we manipulated the retrieval task at test in a between-subjects manipulation (Recall own: *N* = 19; Recall partner: *N* = 19). Additionally, we manipulated the concurrent load during observation within-subjects (No load, Action planning load, Verbal load for 5 cues each). As in Experiments 1 and 2, we will focus on contrasting observed source errors with those predicted by guessing. Since we manipulated concurrent load only during observation of actions, we will focus on the effects of concurrent load on the false retrieval of observed actions in the Recall own task.

### Results

#### Generation Phase

Unlike Experiment 2, there was a main effect of Concurrent load on participants repeating their own actions (No load: *M* = 2.63%, *SD* = 4.15%; Action planning load: *M* = 4.87%, *SD* = 6.09%; Verbal load: *M* = 5.66%, *SD* = 7.28%), *F*(2,74) = 5.02, *MSe* = 0.75, *p* = 0.009, $\eta_{p}^{2}$ = 0.120, with Bonferroni-adjusted comparisons showing that participants repeated more of their own actions in concurrent load conditions (Action planning load, *p* = 0.033; Verbal load, *p* = 0.023) compared to the no load condition. As in Experiment 2, participants more often copied partner’s actions under load, but this effect was not significant here (No load: *M* = 8.68%, *SD* = 6.75%; Action planning load: *M* = 10.66%, *SD* = 7.90%; Verbal load: *M* = 11.05%, *SD* = 7.98), *F*(2,74) = 1.12, *MSe* = 2.45, *p* = 0.33, $\eta_{p}^{2}$ = 0.029. For the analysis of retrieval performance, only those items that had only been performed by one of the participants in the pair were included.

#### Test Phase

We will again first report the conventional analyses for the actions participants report at retrieval. Participants’ responses at retrieval (correct responses, source errors, intrusion errors) were analyzed as multiple 2 Task (Recall own, Recall partner) × 3 Concurrent load (No load, Action planning load, Verbal load) ANOVAs with repeated measures on the second factor.

Correct responses were more frequent in the Recall own than Recall partner task, *F*(1,36) = 41.99, *MSe* = 2.59, *p* < 0.001, $\eta_{p}^{2}$ = 0.538. As in Experiment 2, there was a main effect of Concurrent load, *F*(1.707,61.453) = 9.02, *MSe* = 3.28, *p* = 0.001, $\eta_{p}^{2}$ = 0.200. Pairwise Bonferroni-adjusted comparisons showed that while each of the two load conditions led to fewer correct actions being reported than the control condition (both *p* = 0.001), the two load conditions did not differ from one another, *p* = 1. There was no interaction, *F* < 1.

For source errors, there was no main effect of Task, *F*(1,36) = 1.56, *MSe* = 0.68, *p* = 0.22, $\eta_{p}^{2}$ = 0.041, but a main effect of Concurrent load, *F*(2,72) = 3.52, *MSe* = 1.37, *p* = 0.035, $\eta_{p}^{2}$ = 0.089, with Bonferroni-adjusted comparisons showing that source errors were more frequent in the Action planning load than the No load condition (*p* = 0.047), with the remaining comparisons not significant, *p*s > 0.43. There was no significant interaction, *F* < 1.

Intrusion errors were more frequent in the Recall partner than Recall own task, *F*(1,36) = 8.39, *MSe* = 3.64, *p* = 0.006, $\eta_{p}^{2}$ = 0.189. There was no effect of Concurrent load nor was there an interaction, both *F*s < 1.

**FIGURE 5:**
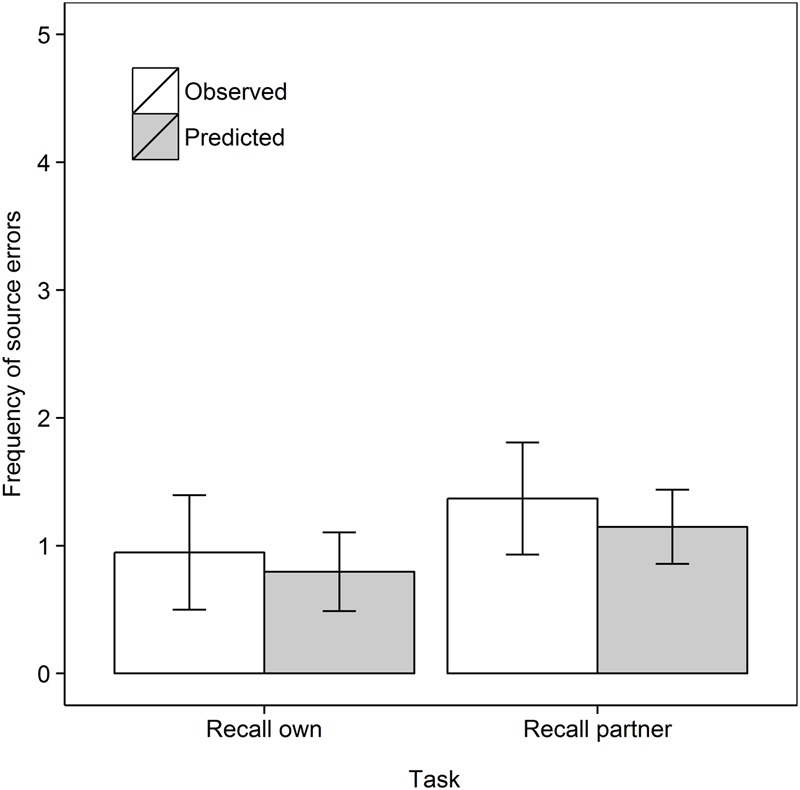
Frequency of observed and predicted source errors in the Recall own and Recall partner task in Experiment 3 for the no concurrent load condition. The error bars are 95% within-subjects confidence intervals.

To turn to our measures of interest, we contrasted the rate of source errors observed versus predicted from guessing as in Experiments 1 and 2. As in Experiment 2, we first compared observed and predicted source errors in the Recall own and Recall partner task, when all actions were encoded without a concurrent load (see **Figure 5**). We analyzed the data with a 2 Task (Recall own, Recall partner) × 2 Data type (Observed, Predicted) mixed ANOVA with repeated measures on the second factor. There was a main effect of Task reflecting that source errors more likely in the Recall partner than Recall own task, *F*(1,36) = 5.27, *MSe* = 0.54, *p* = 0.028, $\eta_{p}^{2}$ = 0.128. However, there was no evidence that source errors were observed more frequently than predicted from guessing, *F*(1,36) = 1.35, *MSe* = 0.49, *p* = 0.25, $\eta_{p}^{2}$ = 0.036, nor was there an interaction, *F* < 1. Separate analyses showed that in fact observed frequencies did not significantly surpass predicted frequencies in either Recall own, *t*(18) = 0.75, *p* = 0.23, *d*_av_ = 0.21 or Recall partner task, *t*(18) = 0.89, *p* = 0.19, *d*_av_ = 0.31, both one-tailed.

**FIGURE 6:**
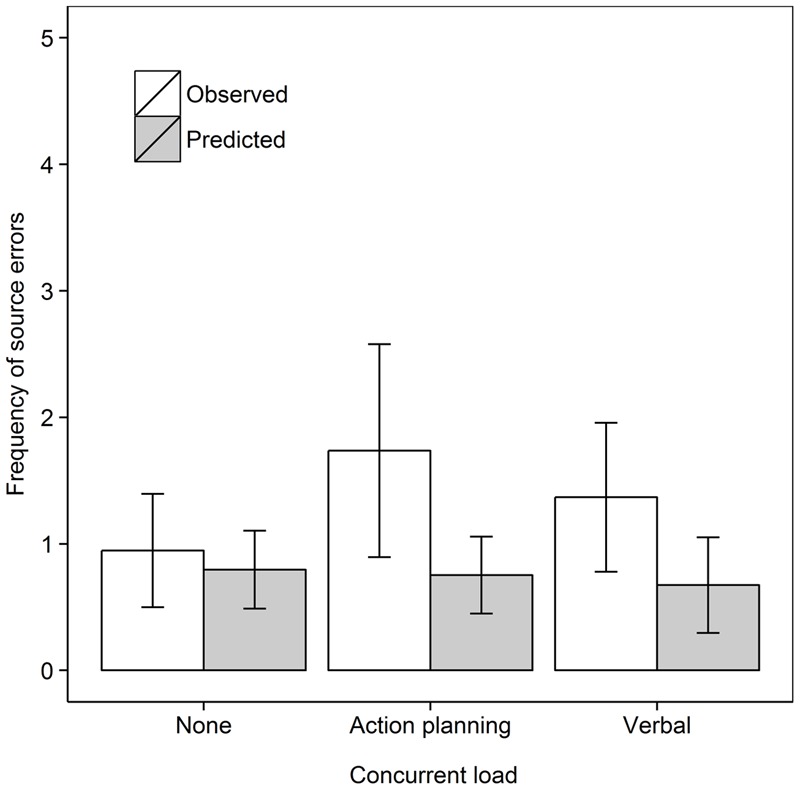
Frequency of observed and predicted source errors in the Recall own task in Experiment 3 for all concurrent load conditions. The error bars are 95% within-subjects confidence intervals.

We next tested the effect of concurrent load on source errors committed in the Recall own task only, as shown in **Figure 6**. We analyzed the data as a 2 Data type (Observed, Predicted) × 3 Concurrent load (No load, Action planning load, Verbal load) repeated measures ANOVA. Source errors were committed more frequently than predicted, *F*(1,18) = 10.16, *MSe* = 1.04, *p* = 0.005, $\eta_{p}^{2}$ = 0.361. There was no evidence for an overall effect of Concurrent load, *F*(2,36) = 2.16, *MSe* = 0.62, *p* = 0.13, $\eta_{p}^{2}$ = 0.107. The interaction was not significant, *F*(2,36) = 2.05, *MSe* = 0.83, *p* = 0.14, $\eta_{p}^{2}$ = 0.102.

In subsequent step-down analyses, we tested whether observed source errors were greater than predicted source errors in every concurrent load condition. Observed frequencies significantly surpassed predicted frequencies in the Action planning load, *t*(18) = 2.61, *p* = 0.009, *d*_av_ = 0.82, and Verbal load condition, *t*(18) = 2.18, *p* = 0.021, *d*_av_ = 0.74, but not in the Control condition, *t*(18) = 0.75, *p* = 0.23, *d*_av_ = 0.21, all comparisons one-tailed.

### Discussion

Experiment 3 shows a similar pattern of data to Experiments 1 and 2, in that participants falsely recalled observed actions as self-performed and self-performed actions as observed to similar degrees. However, in the no load conditions, in contrast to the previous experiments, source errors of either kind did not occur more frequently than guessing in the control condition.

In addition, the motor simulation account predicts that performing a visuospatial or motor task concurrent to observation of actions should decrease source errors compared to observing actions without a concurrent task (e.g., Lindner et al., 2010, 2016), or compared to a task without a visuomotor component, such as the verbal load task used here. Experiment 3 disconfirms this prediction. Source errors were higher than predicted from guessing for both the motor load and the verbal load conditions and were, if anything, more frequent in the verbal load condition.

Thus, Experiment 3 provides interesting data for the impact of monitoring processes on source errors during the recall of action memories. While false retrieval of observed actions as self-performed exceeded guessing in the concurrent load conditions, the same was not true in the control condition. In fact, in the control conditions, source errors committed in either retrieval task did not exceed guessing. This suggests that careful monitoring of the source-appropriateness at recall in the test phase may be able to reduce the source memory error committed at retrieval under certain circumstances.

## General Discussion

### Meta-Analysis of the Three Experiments

The observation inflation effect (Lindner et al., 2010) is the false retrieval of observed actions as being self-performed and has been attributed to motor simulation due to mirror neuron network activation during observation. We tested (1) whether we can conceptually replicate the observation inflation effect with a simpler paradigm that rules out verbal and object-based encoding of the actions, (2) whether there is a complementary, reverse error during the retrieval of partner’s actions, and (3) whether motor system loads during observation reduce the observation inflation effect. So far we have presented results separately for all three experiments. As we pointed out earlier, the imprecision is high (the power low) in some of the single data points, making conclusive interpretation of the overall effect difficult. We therefore integrated the data across experiments into two forest plots (Cumming, 2012) as such a meta-analytic approach should be less affected by noise than the individual studies. Rather than looking at individual comparisons, we can now look at summary effects that contain the effects of the individual comparisons, with more precise effect size estimates contributing more to the summary effect.

**FIGURE 7:**
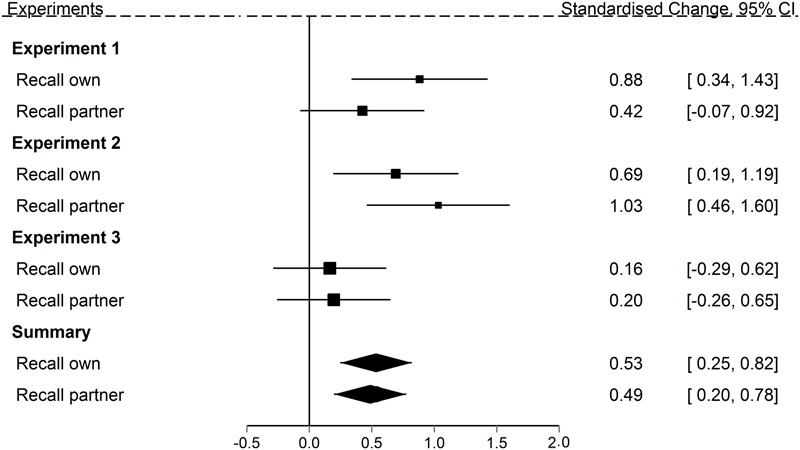
Standardized mean change between observed and predicted source errors committed in Experiments 1 through 3 in both the Recall own and Recall partner task when action were encoded without a concurrent load. Size of the squares represents the weights of the individual comparisons. The error bars are 95% confidence interval. The polygons represent the summary effects (fixed effects) of the Recall own and the Recall partner task, respectively, across all three experiments.

With respect to our first two questions the results are clear. **Figure 7** shows the memory error effects for the control (no load) conditions for all three experiments relative to guessing performance at 0. Summary effect sizes for the Recall own and Recall partner task, given at the bottom of the figure, reveal two clear effects. First, both observation inflation and the reverse error – reporting own actions as those of the partner – occur reliably more often than predicted from guessing. Second, the confidence intervals for the two errors overlap considerably. At present, therefore, there is no evidence to suggest that one form of error is more frequent than the other. We therefore not only successfully demonstrated that the observation inflation effect can be replicated in a simpler recall paradigm that is less vulnerable to effects of verbalization and ambiguous source, but that there is complementary reverse error. This argues against motor activation views of observation inflation, according to which to potential for source-confusion should exist specifically for observed actions, but not self-performed ones.

Our third question was whether a motor system load at encoding would reduce the magnitude of the observation inflation effect. Motor activation views suggest that occupying the motor system would undermine the formation of self-related representations of the partner’s actions, and therefore reduce source errors. We therefore investigated participants’ false recall of their partner’s actions depending upon whether they were under any motor load at encoding or not. The summary statistics in **Figure 7** support two clear conclusions. First, across experiments, there is a robust observation inflation effect regardless of concurrent load. Second, the data do not support the predicted reduction in observation inflation with a motor load: the confidence intervals on the summary statistics of the control and motor load conditions overlap considerably, with the overall pattern suggesting a slight increase in errors with motor load, rather than the predicted decrease.

**FIGURE 8:**
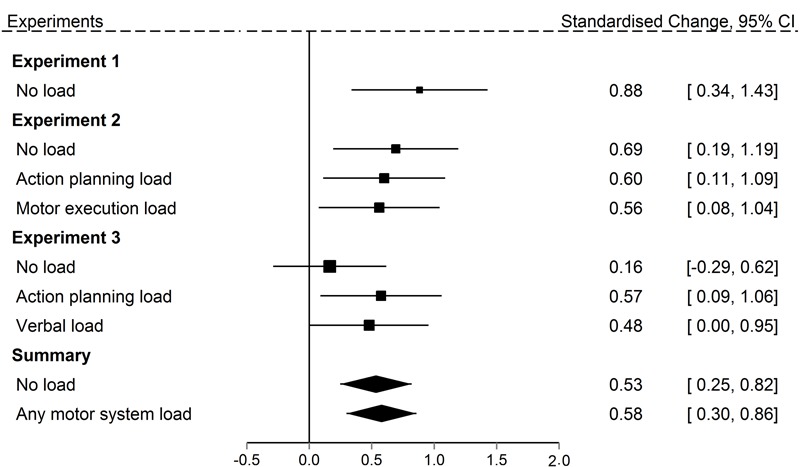
Standardized mean change between observed and predicted source errors committed in the Recall own task in Experiments 1 through 3 for all concurrent load conditions. Size of the squares represents the weights of the individual comparisons. The error bars are 95% confidence interval. The polygons represent the summary effects (fixed effects) of the No load conditions and the Motor system load conditions (Action planning load and Motor execution load).

Finally, **Figure 8** allows closer comparison of the results in Experiment 3 relative to the prior experiments. The main difference between Experiments 3 and 2 was the change in retrieval task instruction that required participants to carefully examine the actions they were reporting. Rather than examining Experiment 3 in isolation, we can now ask if the change in retrieval task instruction replicated the effect of Experiment 2. We used a Bayesian approach, employing the Dienes (2008) protocol and using the Jeffreys (1961) guidelines for interpreting Bayes Factors. The advantage of the Bayesian approach to data analysis is that it allows specification of an exact expected effect size based on prior information (in this case, Experiment 2) compared to a null effect (source errors do not exceed guessing), and provides an estimate of the extent to which the evidence (Experiment 3 data) support either of these two hypotheses. The evidence is judged to be inconclusive if neither hypothesis is clearly favored due to, for example, a lack of power in the experiment.

We first compared the no load condition in both the Recall own and Recall partner retrieval task from Experiment 3 to the matching condition in Experiment 2. The prior for the Recall own task was defined by the effect in Experiment 2 with a half-normal distribution, i.e., one-tailed, with *M* = 0, *SD* = 1.28. The data was defined by the effect in Experiment 3 with a sample mean of *M* = 0.15 and *SE* = 0.20. The prior for the Recall partner task was defined by the effect in Experiment 2 with a half-normal distribution, i.e., one-tailed, with *M* = 0, *SD* = 2.04. The data was defined by the effect in Experiment 3 with a sample mean of *M* = 0.22 and *SE* = 0.25. This resulted in Bayes Factors of 0.32 for the Recall own and 0.29 for the Recall partner task. This means that the data in Experiment 3 provide 3 and 4 times more evidence, respectively, in favor of a null memory error effect than in favor of a replication of the effect in Experiment 2. This suggests that careful monitoring reduced memory errors effect when recalling actions observed without a load.

We next compared the action planning load condition in the Recall own task across the two experiments. The prior was defined by the effect in Experiment 2 with a half-normal distribution, i.e., one-tailed, with *M* = 0, *SD* = 0.95. The data was defined by the effect in Experiment 3 with a sample mean of *M* = 0.98 and *SE* = 0.38. When observed actions were encoded under concurrent action planning load, the size of the memory error in the Recall own task in Experiment 3 is more compatible with the size of the effect in the matching condition in Experiment 2 than the null, Bayes Factor = 15. This means that the memory error effect observed in the action planning condition in Experiment 3 is 15 times more compatible with the memory error effect in Experiment 2 than with a null memory error effect. This suggests that careful monitoring at retrieval can reduce source errors during recall, but only does so when participants were not engaged in a secondary task during the encoding of actions. We will return to the theoretical implications of this later.

### Does the Observation Inflation Effect Generalize?

The first aim of this series of studies was to test if the observation inflation effect reported by Lindner et al. (2010) generalizes to a different experimental design, retrieval task and with different action stimuli. Across three experiments, we have shown a robust effect of observed actions being falsely retrieved as self-performed actions.

This suggests that the observation inflation effect is not merely an effect of verbalisable action phrases, but can also occur with non-verbalisable, non-object-directed actions. Additionally, it shows that the observation inflation effect is not solely a result of the experimental design or retrieval task. In this series of experiments, we observed robust evidence of observed actions leading to false claims of self-performance in two versions of a free recall paradigm.

### What Is the Role of the Motor Component in Source Confusion?

Across three experiments, we tested whether motor activation during action observation underlies false claims of observed actions as self-performed. We failed to find evidence for this account.

First, an account based on internal replicas of observed actions (Craighero et al., 1999, 2002) does not explain why self-performed actions were reported as “observed” in all three experiments, since motor activation during performance always points toward self-performance, and no conflict between self- and other performance should arise. Moreover, if mirror neuron activation was fundamentally responsible for later false memories of self-performance, such false memories should be disrupted when observers’ motor systems are occupied with a motor task at the time of observation. However, direct manipulations of the extent to which the observed actions could be mirrored at encoding showed no evidence for the expected reduction in subsequent source errors, in either Experiment 2 or 3. In contrast, Lindner et al. (2016) found a reduction in the effect of observation on false and correct memories of performance when participants performed incongruent actions during observation relative to when they performed congruent actions (either temporally aligned or shifted), suggesting a possible disruption of motor simulation. We could not confirm that claim for false memories specifically when testing concurrent action execution of incongruent actions against both an observation-only condition and against a non-motor verbal load condition in our paradigm.

Of course, while our manipulations aimed to disrupt basic motor execution and higher level action planning, it is possible that neither sufficiently reduced motor simulation. However, concurrent motor action, with very similar procedures, has been shown to impair perception of observed actions (Vetter and Wolpert, 2000; Hamilton et al., 2004; Zwickel et al., 2007) and the acquisition of motor skills during imitation and observation learning (Bach et al., 2014b), and observation of actions has been shown to influence execution of actions (Kilner et al., 2003; Bach and Tipper, 2007; Press et al., 2011), suggesting a bidirectional influence between action perception and execution (for reviews, see Müsseler, 1999; Schütz-Bosbach and Prinz, 2007; Zwickel and Prinz, 2012; Avenanti et al., 2013; Campbell and Cunnington, 2017). Indeed, we found that both low-level and high-level motor system load affected the duplication of a partner’s actions in the encoding task as well as correct recall of observed actions at test, confirming that our load manipulation was generally effective.

A second possibility is that we were successful in disrupting the encoding of the motor component of observed actions but additionally disrupted the encoding of other memory components (e.g., due to cognitive load induced by the additional task). While the disruption of the encoding of the motor component is associated with a decrease in source errors, this may have been counteracted by an increase in source errors due to the overall cognitive load at encoding (Craik, 2014). However, we would have then expected to see a difference in the number of source errors between the different concurrent load conditions. Under such an account, source errors in the more cognitively demanding action planning load task should have been more frequent than in the motor execution task in Experiment 2 and source errors should have been less frequent in the more motorically taxing action planning load task than the equally cognitively demanding verbal load in Experiment 3. We found no evidence for this pattern of effects.

What do our results mean for the observation inflation effect in the observation inflation paradigm by Lindner et al. (2010)? Our results certainly suggest, as shown by these prior studies, that performing and observing actions leads to source confusion about actions having been performed or observed. However, we did not find any evidence for motor encoding of observed actions impacting the frequency of observed actions falsely remembered as self-performed. Indeed, Lindner et al. (2016) acknowledge that while motor simulation may occur during observation, additional processes such as consolidating information from different sources of memory are necessary to account for the inflation effect.

### Can the Source Monitoring Framework Account for the Data?

The source monitoring framework (Johnson et al., 1993) suggests that the source of any given information is not specifically encoded but inferred from qualitative features encoded alongside the item information at test. We propose that the source errors observed here and in the observation inflation paradigm can be accounted for in this framework. In such a view, actions do not have a special status, but are remembered just like any other event. As such, they are compatible with recent ideomotor accounts of action and action observation, which argue that actions are learned, stored and planned on the perceptual level, in terms of the perceptual effects that go along with them (e.g., Hommel et al., 2001), such as the trajectories they produce or the proprioceptive, visual and auditory feedback they generate.

A source monitoring account can explain all features of the data in our experiments and has been previously suggested to account for false memories of self-performance after visualization of actions (for a discussion, see Henkel and Carbuto, 2008; Lindner and Henkel, 2015; also see Leynes and Kakadia, 2013). Firstly, a source monitoring account can explain why participants not only misremember observed actions as performed but also performed actions as observed. Performed and observed actions both create memories of events that share similarities such as the body parts used, their trajectories and the manner of performance. These commonalities predict not only source errors in the recall own task, but a general confusion about the origin of encoded actions that would affect both tasks equally, and more so if these disambiguating aspects are not encoded. Lindner et al. (2016) suggest that even if motor simulation occurs, further processes are necessary to result in false claims of self-performance after observation. It is plausible that evaluation of motor traces in addition to verbal or cognitive traces is that consolidation process.

Secondly, our results are compatible with the predictions of a source monitoring account by showing a decrease in source confusion when participants evaluate all qualitative features of individual memories systematically. Indeed, the retrieval task instructions we used in Experiment 3 did eliminate source errors. This suggests that allowing participants to withhold remembered items from report is an effective strategy of decreasing the reports of source errors when retrieving action events by preventing participants from neglecting to consider the source of recalled memories. This is in line with effects that showed that source performance can be drastically affected by the instruction and decision required at test (Dodson and Johnson, 1993; Marsh et al., 1997; Marsh and Hicks, 1998). Interestingly, finally, that same retrieval task manipulation failed to show the same elimination of observation inflation when actions had been encoded under concurrent load. Under source monitoring account predictions, any distraction that prevents the encoding of qualitative features of items should result in a poorer source memory trace, as fewer source-relevant features might be encoded. Our data suggest that concurrent load prevented the encoding of source-diagnostic information for individual actions to such a degree that even systematic consideration of source at test was unable to overcome that impairment.

There is a possibility that our paradigm may have led to increased source confusion on the basis of participants visualizing themselves from a third-person perspective (Leynes and Kakadia, 2013). However, we deem it unlikely that perspective taking would have fundamentally given rise to magnitude of source confusion we observed. Our instructions explicitly discouraged participants to imagine their own performance from the other person’s perspective. Given that perspective taking is cognitively demanding (e.g., Bull et al., 2008; Qureshi et al., 2010; Surtees et al., 2016), participants would therefore not spontaneously engage in it, if not motivated by such task demands.

## Conclusion

This series of studies demonstrates that while we cannot comment on whether observed actions are mirrored at encoding, we could not find any evidence for mirroring translating into false memories of action performance after a delay. Given that a source monitoring account can account for source confusion of verbal items and action events as well as the variety of patterns in our data, it is at this stage not apparent what additional explanatory contribution a mirror neuron network account could make to an understanding of source memory for actions, at least for tasks and actions such as ours.

## Author Contributions

Substantial contributions to the conception or design of the work; or the acquisition, analysis, or interpretation of data for the work: NL, TH, and PB. Drafting the work or revising it critically for important intellectual content: NL, TH, and PB. Final approval of the version to be published: NL, TH, and PB. Agreement to be accountable for all aspects of the work in ensuring that questions related to the accuracy or integrity of any part of the work are appropriately investigated and resolved: NL, TH, and PB.

## Conflict of Interest Statement

The authors declare that the research was conducted in the absence of any commercial or financial relationships that could be construed as a potential conflict of interest.
